# Description of color/race in Brazilian biomedical research

**DOI:** 10.1590/S1516-31802012000200008

**Published:** 2012-04-03

**Authors:** Teresa Veronica Catonho Ribeiro, Luzitano Brandão Ferreira

**Affiliations:** I Pharmacist and Postgraduate student, Universidade de Brasília (UnB), Brasília, Brazil.; II MD, PhD. Associate Professor, Faculty of Health Sciences, Centro Universitário de Brasília (UniCEUB), Brasília, Brazil.

**Keywords:** Ethnic groups, Socioeconomic factors, Public health, Health services research, Biomedical research, Grupos étnicos, Fatores socioeconômicos, Saúde pública, Pesquisa em serviços de saúde, Pesquisa biomédica

## Abstract

**CONTEXT AND OBJECTIVE:**

Over recent years, the terms race and ethnicity have been used to ascertain inequities in public health. However, this use depends on the quality of the data available. This study aimed to investigate the description of color/race in Brazilian scientific journals within the field of biomedicine.

**DESIGN AND SETTING:**

Descriptive study with systematic search for scientific articles in the SciELO Brazil database.

**METHODS:**

A wide-ranging systematic search for original articles involving humans, published in 32 Brazilian biomedical scientific journals in the SciELO Brazil database between January and December 2008, was performed. Articles in which the race/ethnicity of the participants was identified were analyzed.

**RESULTS:**

In total, 1,180 articles were analyzed. The terms for describing race or ethnicity were often ambiguous and vague. Descriptions of race or ethnicity occurred in 159 articles (13.4%), but only in 42 (26.4%) was there a description of how individuals were identified. In these, race and ethnicity were used almost interchangeably and definition was according to skin color (71.4%), ancestry (19.0%) and self-definition (9.6%). Twenty-two races or ethnicities were cited, and the most common were white (37.3%), black (19.7%), mixed (12.9%), nonwhite (8.1%) and yellow (8.1%).

**Conclusion:**

The absence of descriptions of parameters for defining race, as well as the use of vague and ambiguous terms, may hamper and even prevent comparisons between human groups and the use of these data to ascertain inequities in healthcare.

## RESUMO

**CONTEXTO E OBJETIVO:**

Nos últimos anos os termos raça e etnia têm sido usados para verificar iniquidades em saúde pública. Entretanto, o uso dos termos depende da qualidade dos dados disponíveis. O presente estudo teve como objetivo verificar a descrição de cor/raça em revistas científicas brasileiras da área biomédica.

**TIPO DE ESTUDO E LOCAL:**

Estudo descritivo com busca sistemática de artigos científicos da base de dados SciELO Brasil.

**MÉTODOS:**

Foi realizada uma ampla busca sistemática de artigos originais envolvendo seres humanos, publicados em 32 revistas científicas biomédicas brasileiras da base de dados SciELO Brasil, publicados no período de janeiro a dezembro de 2008. Foram analisados artigos em que as raças/etnias dos participantes da pesquisa foram identificadas.

**RESULTADOS:**

Ao todo foram analisados 1.180 artigos. Os termos para descrever a raça ou etnia foram frequentemente ambíguos e vagos. A descrição da raça ou etnia ocorreu em 159 (13,4%) artigos, mas somente em 42 (26,4%) havia a descrição de como os indivíduos foram identificados. Nestes, raça e etnia foram usados praticamente como sinônimos e a definição ocorreu pela cor da pele (71,4%), ancestralidade (19,0%) e autodefinição (9,6%). Foram citadas 22 raças/etnias, sendo as mais comuns a branca (37,3%), a negra (19,7%), a parda (12,9%), a não-branca (8,1%) e a amarela (8,1%).

**CONCLUSÃO:**

A ausência de descrição de parâmetros para definir raça, bem como termos vagos e ambíguos, pode dificultar e mesmo inviabilizar a comparação de grupos humanos e a utilização desses dados para verificar iniquidades em saúde.

## INTRODUCTION

It is a well-established fact nowadays that there is no scientific basis for the human species to be categorized into races and that this classification is inadequate for describing the genetic variation of our species.^1^ It is also known that the word “race” can lead to stereotypes and prejudice. However, these definitions have great potential value in healthcare, especially in multiethnic societies,^2^ for revealing and reversing inequities.^3^

The use that can be made of information on race depends on the quality of the data available. Inadequate description of race or ethnicity in biomedical records may lead to heterogeneous terminology and inconsistent categorization, which complicates or even prevents the use of these data in uncovering inequities in healthcare.^4^

In Brazil, the importance of discussing the definition of race in biomedical research stems from the use of these variables by government health officials, on the one hand, and the small number of related scientific articles, on the other. However, there has not been any in-depth discussion of theoretical and practical problems relating to this subject.^5^

## OBJECTIVE

The objective of this study was thus to investigate the use of the concept of race in Brazilian biomedical journals.

## METHODS

A comprehensive search for original articles involving humans, published in 32 biomedical journals in the Brazilian SciELO database, was performed. The latter was chosen because it hosts the most important Brazilian scientific journals (mostly in Portuguese); because it allows free access to each issue of each journal, including to full texts of articles; and because it is a source of data for Brazilian biomedical researchers, professionals and students.

All studies published between January and December 2008 were analyzed. The inclusion criteria were that the articles needed to have been published in biomedical journals and consist of studies involving humans. Editorials, letters, commentaries, systematic reviews and articles that did not involve humans were excluded.

The initial selection of articles was performed by reading their titles and then their abstracts. Once it was ascertained that the article was a study on humans, reading of the methodology, results and discussion followed. Information was collected from the scientific articles through a predesigned and pretested form. The information sought comprised the following: how the research participants were described (gender, age, marital status, religion, socioeconomic status, educational background and color, race or ethnicity); whether any definition of the participants’ color, race or ethnicity was given, and if so, what the criteria were for that definition and whether they were explained in the study.

The journals analyzed were: Acta Cirurgica Brasileira, Acta Ortopedica Brasileira, Acta Paulista de Enfermagem, Anais Brasileiros de Dermatologia, Arquivo Brasileiro de Cardiologia, Arquivo Brasileiro de Endocrinologia e Metabologia, Arquivo Brasileiro de Gastroenterologia, Arquivos de Neuro-Psiquiatria, Brazilian Journal of Medical and Biological Research, Cadernos de Saúde Pública, Genetics and Molecular Biology, Jornal Brasileiro de Pneumologia, Jornal de Pediatria, Revista Brasileira de Ciências Farmacêuticas, Revista Brasileira de Cirurgia Vascular, Revista Brasileira de Enfermagem, Revista Brasileira de Epidemiologia, Revista Brasileira de Fisioterapia, Revista Brasileira de Ginecologia e Obstetrícia, Revista Brasileira de Hematologia e Hemoterapia, Revista Brasileira de Medicina do Esporte, Revista Brasileira de Ortopedia, Revista Brasileira de Psiquiatria, Revista Brasileira de Reumatologia, Revista Brasileira de Saúde Materno Infantil, Revista da Associação Médica Brasileira, Revista da Escola de Enfermagem da USP (Universidade de São Paulo), Revista da Sociedade Brasileira de Medicina Tropical, Revista de Psiquiatria Clínica, Revista de Saúde Pública, São Paulo Medical Journal and Saúde e Sociedade.

## RESULTS

A total of 1,180 articles from 32 Brazilian biomedical journals were analyzed. The most frequent criteria used to describe the research participants were: gender (87.2%), age (82.9%), education (20.9%), socioeconomic status (15.2%), race (13.4%), marital status (12.4%) and religion (1.7%).

Color, race or ethnicity was one of the parameters used to describe the study participants in 159 papers (13.4%). In 117 (73.6%) of these articles, there was a description of how individuals were defined according to their different races or ethnic groups, but in only 42 (26.4%) was there a description of what the parameters or criteria for classifying individuals were. In these 42 articles, the terms color, race and ethnicity were used interchangeably. Among them, race or ethnicity was defined by individuals’ skin color in 32 (76.2%), by ancestry in six (14,3%) and through individuals’ self-definition in four (9.5%).

In total, 22 different color/races were described in the scientific articles reviewed. The number of races defined in each study ranged from one to five. Twenty-six articles (16.3%) defined only one race, 73 (45.9%) defined two, 37 (23.3%) defined three, 15 (9.4%) defined four and eight (5.1%) defined five different races in their classifications.

The most prevalent races in the scientific articles were: white (142; 37.3%), black (75; 19.7%), mixed race (49; 12.9%), nonwhite (31; 8.1%), yellow (31; 8.1%). Other “races” cited were mulatto, Native American, Caucasian, half-blood, Afro-descendent, Euro-descendent, white of non-Germanic ancestry, white of Germanic ancestry, brown, swarthy, Asian, non-Caucasian, non-Afro-descendent, Japanese-descended, Brazilian-descended and *quilombola*.

## DISCUSSION

Racial categories are created and maintained within sociopolitical contexts and change meaning over time. Scientific data are often embedded in specific political agendas, even without knowledge of their meaning and repercussions. Thus, the definition of race in scientific research, especially in biomedicine, needs to be well defined, justified and properly discussed.

In this study, research participants’ race was described in only a few of the articles (13.5%) analyzed. Parameters for categorizing individuals of different races were also only defined in a minority of studies (25.5%). Description of race or ethnicity, especially in studies that ascertain individuals’ access to and use of healthcare services, may be very useful in revealing and reversing inequities involving different population groups.

Among the articles that described the criteria used to identify individuals of different races, the vast majority (75%) used skin color as a parameter. Using such data, especially in clinical research, is controversial and should be done very carefully, as research shows that in Brazil there is no clear correlation between phenotypic characteristics, especially skin color, and individuals’ genomic ancestry.^6^

Nonetheless, it is erroneous to conclude that if “race” is not a matter of biological classification but is constructed and reinforced by social norms, then it is both unrealistic and worthless in scientific research.^5^ Biomedical scientists tend to believe that races are neutral descriptors of groups of individuals,^7^ yet this definition represents a racialization process for many patients, with potentially harmful effects, particularly for people who have experienced inequities in healthcare.^8^ When data on race is gathered, research participants should, whenever possible, have the opportunity to identify their own races and be informed that these identities will constitute variables for research and comparison with other racial groups.^9^ In the present study, a small number of the studies that informed how the classification was obtained used racial self-definition as a parameter.

In total, 22 different races or ethnicities were used in the scientific articles reviewed. The number of races defined in the studies ranged from one to five. The terms used to categorize individuals were often vague, such as Asian, dark, swarthy or Brazilian-descended; or were ambiguous, such as “white”, “Caucasian” or “Eurodescendent”. These terms may underestimate certain populations and/or group individuals with different backgrounds, culture and ancestry together in the same denomination.

It is especially important to represent minorities and vulnerable populations in clinical research, in situations where disparities occur in the access to and use of services. However, racial classifications for minorities and vulnerable populations are often forgotten, or such individuals may be placed, with other populations, in categories that may prove unhelpful. In some studies, individuals were racially classified only as “white” or “nonwhite”, which may impair recognition of inequities in certain minority groups.

The findings from this study are similar to those reported in other studies that found the same categorization of individuals according to race or ethnicity. In an analysis on biomedical research supported by the National Cancer Institute of the United States National Institutes of Health, it was not clear what the researchers meant when they used the term race or ethnicity in their inquiries.^7^ The participants in several biomedical research studies were not adequately described in relation to race or ethnicity, even in situations where there were disparities in healthcare.^8^

Even in scientific journals within the fields of epidemiology and public health, these terms are not well defined. Comstock et al.^10^ conducted a review of articles published in scientific journals on American public health. They found that 77% of the articles referred to race or ethnicity, yet the researchers often did not establish the context within which these variables were used, or the methods for defining races, and did not discuss the study results based on these classifications. This variability probably reflects the ambiguity and lack of consensus regarding the definition and categorization of race/ethnicity among researchers.

There are no standards to define race in biomedical research and it may not be possible to reach such a consensus. However, it is possible for researchers to record and attentively discuss why race is being used, how it is being assessed and what results these studies may imply.^10^ Studies that attempt to correlate race and health should seek to understand the vulnerability of certain segments of the population and the extent to which ethnic/racial status constrains or facilitates exposure to health hazards.^5^ There is also a need to understand not only the limitations of race and ethnicity as epidemiological variables and the risks that such classifications may entail, but also the power that such tools may provide, especially within the field of public health, through revealing and reversing inequities in individuals’ access to and use of healthcare services.

## CONCLUSION

The absence of descriptions of the parameters used to define race and ethnicity in the vast majority of cases in which these variables are used, as well as the use of vague and ambiguous terms, may hamper and even prevent comparisons between human groups and the use of these data to ascertain inequities in healthcare.
